# Viruses and mobile elements as drivers of evolutionary transitions

**DOI:** 10.1098/rstb.2015.0442

**Published:** 2016-08-19

**Authors:** Eugene V. Koonin

**Affiliations:** National Center for Biotechnology Information, National Library of Medicine, National Institutes of Health, Bethesda, MD 20894, USA

**Keywords:** evolutionary transitions, mobile genetic elements, parasites, viruses, antivirus defence, host–parasite coevolution

## Abstract

The history of life is punctuated by evolutionary transitions which engender emergence of new levels of biological organization that involves selection acting at increasingly complex ensembles of biological entities. Major evolutionary transitions include the origin of prokaryotic and then eukaryotic cells, multicellular organisms and eusocial animals. All or nearly all cellular life forms are hosts to diverse selfish genetic elements with various levels of autonomy including plasmids, transposons and viruses. I present evidence that, at least up to and including the origin of multicellularity, evolutionary transitions are driven by the coevolution of hosts with these genetic parasites along with sharing of ‘public goods’. Selfish elements drive evolutionary transitions at two distinct levels. First, mathematical modelling of evolutionary processes, such as evolution of primitive replicator populations or unicellular organisms, indicates that only increasing organizational complexity, e.g. emergence of multicellular aggregates, can prevent the collapse of the host–parasite system under the pressure of parasites. Second, comparative genomic analysis reveals numerous cases of recruitment of genes with essential functions in cellular life forms, including those that enable evolutionary transitions.

This article is part of the themed issue ‘The major synthetic evolutionary transitions’.

## 1. Introduction

As forcefully propounded by Charles Darwin [1] and solidified in the neo-Darwinian synthesis [2,3], evolution in general proceeds gradually through small heritable changes (mutations, in modern parlance). Yet it is abundantly clear that the diversity of life on Earth as we know it today must have been shaped, on top of the gradual change, by numerous, dramatic innovations that appear to leave gaps between levels of biological organization. In their seminal papers and monograph, Maynard Smith and Szathmáry [4–8] noted such innovative evolutionary transitions and distinguished between major and minor transitions. The term ‘transition’ should not be taken to mean that organisms at a new level of complexity replace the pre-existing, simpler ones but rather that the simpler and more complex levels of biological organization come to coexist upon the transition. In the latest incarnation of the concept [8], Szathmáry lists seven major transitions, from the emergence of protocells to the advent of human societies with language (table 1). In addition, many minor transitions can be delineated.

**Table 1.** The major evolutionary transitions and contributions of mobile genetic elements (MGE).

major transition	from	to	contribution of MGE
1	small, virus-like replicators (‘naked genes’)	stable, compartmentalized ensembles of replicators; protocells	— the primordial pool of replicators: virus-like genetic entities — emergence of parasites with first replicators — pressure of parasites leads to compartmentalization — pressure of parasites leads to separation of templates and catalysts • origin of translation/proteins • origin of DNA, dedicated information storage media — diversification of replication/expression strategies — origin of large, chromosome-scale DNA genomes as means for fixation of cooperating ensembles — origin of MGE integration — origin of defence systems — origin of cells creates barriers to MGE spread, triggers origin of viruses
2	protocells	prokaryotic cells	
3	prokaryotic cells	eukaryotic cells	— massive transfer of MGE, primarily Group II introns, from endosymbiont to host genome, proliferation of transferred MGE in the host genome — origin of introns, snoRNA, PRP8 from Group II introns — selective pressure for defence against introns drives the evolution of spliceosome, nucleus, nonsense-mediated decay (NMD), ubiquitin network — chromosome linearization as defence against deletions caused by intron recombination; exaptation of Group II intron RT for the telomerase function
4	heterotrophic eukaryotic cells	autotrophic (photosynthetic) eukaryotic cells	? (contribution of introns from the symbiotic cyanobacterium?)
5	unicellular life forms	multicellular life forms (animals, plants, fungi, brown algae)	— multiple, independent occasions of concomitant origin of simple, aggregative multicellularity and programmed cell death (PCD) under pressure of parasites
6	non-social animals	eusocial animals	? (viral infections? Bursts of MGE transposition?); likely role of non-viral parasites
7	societies of eusocial animals—no natural language	societies with natural language	?

**Figure 1.**
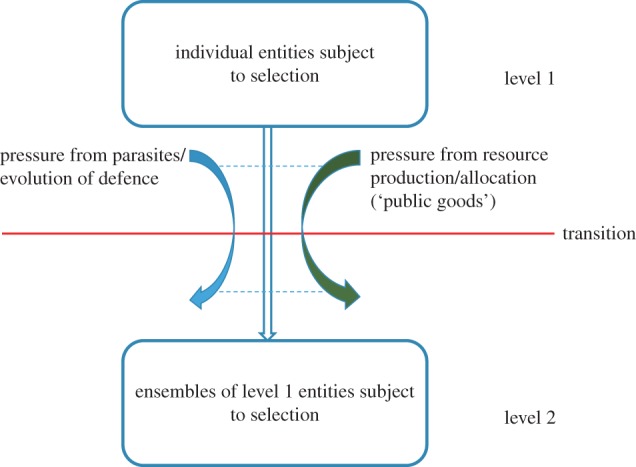
Evolutionary transitions. (Online version in colour.)

The evolutionary transitions are not arbitrarily chosen, even in important innovations that occurred in the course of the history of life. Following the earlier discussion by Queller [9], Szathmáry emphasizes two dimensions of a transition: ‘The Acquisition of Inheritance Characteristics’ and ‘Cooperators since Life Began’ [8]. The second aspect appears to be the one that is easier to describe in specific terms and pin down for each individual transition. Indeed, the key conceptual insight of Maynard Smith and Szathmáry is that each of the transitions involves the emergence of a new, complex selectable entity—in other words, a new level of selection that acts on ensembles of entities from the previous level (figure 1). The origin of multicellular organisms, which occurred on at least three independent occasions in the course of the evolution of eukaryotes, is perhaps the most obvious case in point whereby selection at the level of individual cells is supplanted by selection on cell collectives. One could argue that evolution of complex systems in general involves a transition to selection at a higher level, that of an ensemble of entities, such as a group of genes encoding a particular set of proteins, from the selection at the lower level of individual entities (genes) [10,11]. Thus, the concept of evolutionary transitions, in principle, can be perceived as a general theory for the evolution of complexity [12]. In a higher abstraction plane, this concept is linked to the earlier theory of metasystem transitions, defined as emergence of metasystems via integration of originally independent components, which was developed by Turchin in 1970 [13].

Each major transition appears to have involved evolution of mechanisms that control selection at the preceding, lower level of organization. Reversion to the lower level of selection can be deleterious to the higher level, complex entities as obviously illustrated by cancer. The evolutionary factors underlying the transitions are not well understood in general although some strong candidates, such as mitochondrial endosymbiosis for the origin of eukaryotes, are apparent.

All or nearly all cellular life forms harbour diverse genetic parasites including transposons, plasmids, viruses and other selfish elements [14]. Parasite–host coevolution is a major aspect of all evolution of life [15–19]. In large part, this coevolution takes the form of the incessant arms race during which the cellular hosts evolve multiple, in some cases highly elaborate mechanisms of resistance and defence to which the parasites respond by evolving counter-defence systems [20–23]. However, cooperation between hosts and parasites complements the arms race. Parasitic genetic elements can provide benefits to the host by protecting the host from superinfection, but also through exaptation of genetic material of the selfish elements for host functions [24,25]. Sequences derived from mobile genetic elements (MGE) constitute large fractions of the genomes of many eukaryotes, e.g. up to 90% of some plant genomes and are considered to be important drivers of genome evolution [26–32]. Mobilization of MGE can be beneficial by promoting diversification, especially under stress, but can also be deleterious [33,34]. A major role of viruses in the host biology has also been demonstrated in prokaryotes as illustrated by gene transduction, in particular the transfer of photosystem genes by cyanophages [35,36], the use of defective prophages as vehicles for horizontal gene transfer (HGT) known as gene transfer agents [37], and more generally by the recruitment of viral genes for diverse host functions [38].

The relationships between genomic parasites and their hosts encompass a wide range of ‘selfishness’, i.e. the parasites form a broad spectrum with respect to the cost they incur on the host [39–41]. At one end of the spectrum are benign elements that are incapable of autonomous replication and only replicate with the host genome so that the cost of the parasite is limited to the near negligible cost of its replication. At the opposite end are lytic viruses that replicate to extremely high copy numbers and rapidly kill the host. Diverse selfish elements with various degrees of autonomy fit between these extremes including temperate viruses, transposons and plasmids. Multiple evolutionary links (i.e. shared genes) connect selfish elements that differ in terms of the cost to the host such as lytic viruses and transposons [42]. Moreover, the same element often alternates between different lifestyles, e.g. between low-cost proviruses and high-cost lytic viruses, as in the thoroughly studied cases of lysogenic bacteriophages [43–45] and animal retroviruses [46,47], or between a virus and a plasmid [48]. Most cellular organisms co-host different classes of parasitic selfish elements, resulting in complex ecosystems of interacting replicators.

Numerous environmental studies confidently indicate that viruses are the most abundant biological entities on Earth, with the number of virus particles in environments as diverse as seawater, soil and animal guts exceeding the number of cells by one to two orders of magnitude [49–52]. Given this dominance of viruses in the biosphere, the invariable association of viruses and/or other selfish elements with cellular life forms (encapsulated in the virocell concept of Forterre [53,54]) and the emergence of parasites in theoretical and experimental models of replicator systems, a major role of these elements in all stages of the evolution of life appears self-evident.

Here, I present evidence and argument that selfish elements make key contributions to evolutionary transitions at least at two levels. First, the parasite–host arms race leads to increased organizational complexity of biological systems, i.e. serving as a catalyst of evolutionary transitions. Second, viruses and mobile elements contribute specific genes that play major roles in the emergence of coherent wholes at a new level of complexity. Thus, host–parasite coevolution appears to be one of the key driving factors of evolutionary transitions along with public goods sharing (resource allocation). The discussion below is limited almost entirely to the earlier major transitions that involve innovations at the molecular and cellular levels, with the origin of eusocial animals and the origin of the human society that play out at the organismal level being largely outside the scope of this article.

## 2. The first and second major transitions: from pre-cellular communities of replicators to protocellular reproducers

According to Szathmáry [8], the first major transition in the history of life involved the origin of the protocells, whereas the second transition consisted of the origin of translation and prokaryote cells (table 1). Staging of these processes is less than obvious, e.g. it is unclear whether the formation of chromosomes predates (as assumed by Szathmáry) or postdates the origin of translation. Therefore, here I address these early stages of evolution as a single, complex transition.

The apparent presence of genomic parasites at all levels of biological organization and in association with all cellular life forms suggests the possibility that such parasites are inherent to life. Indeed, parasites consistently emerge in theoretical models of simple replicator systems [5,55–58]. Without going into mathematical details, it is intuitively obvious that in any replicator system which depends on a resource that limits the replication rates, such as a replicase, cheaters will emerge that exploit the resource without producing it. Indeed, it can be expected that the endgame in the evolution of cheaters is a minimal genome consisting solely of signals for replicase recognition and utilization. That this outcome is realistic in a simple replicator system is indicated by the results of the early, classical ‘*in vitro* Darwinian’ experiments of Spiegelman and co-workers [59–61] (subsequently repeated with various modifications in several laboratories) in which the genomic RNA of bacteriophage Qβ was incubated with the phage replicase for a limited time. After multiple serial transfers between test tubes, the population had been found to consist of a mini-genome that had lost all protein-coding genes and only retained the signals for the interaction with the replicase [62].

The pre-cellular stage of biological evolution (when referring to a pre-cellular stage, I do not imply lack of compartmentalization, rather only the lack of organization typical of modern cells) can be plausibly envisaged as a viral-like state. In this model, the subjects of evolution (that is, selection and random drift) were small; genetic elements in the size range of the extant RNA viruses, i.e. approximately 1–30 kb, that were partially or fully selfish [57,63–65] (table 1). Given that cells have not yet evolved, it is probably inappropriate to speak of viruses with respect to this stage of evolution but it seems undeniable that modern-type cellular life was preceded by pools of genetic elements that were virus-like in terms of characteristic size and partial autonomy [14]. Regardless of the molecular details, the differentiation into cooperators and cheaters emerged already at the onset of the primordial genetic pools.

A striking theoretical insight is that a spatially homogeneous population of replicators is prone to be overtaken by cheaters (parasites) and hence is generally doomed to collapse [66,67]. The only path to stability for a population of replicators in the face of the parasite onslaught appears to be compartmentalization: parasites can be eliminated from a population or at least kept in check if the rate at which the parasite is transferred to new hosts is insufficient to compensate for the loss of the parasite [58,66,68]. Compartmentalization creates barriers for the spread of parasites and conditions for cooperation between non-parasitic replicators that can result in an ensemble of cooperators outcompeting the parasites locally and stabilizing the coevolving system globally [67,69]. Thus, parasites promote group selection among cooperators, which paves the path to the evolutionary transition [4,55]. Accordingly, in pre-cellular ensembles of virus-like elements, there would be selection for increasing genome size by joining segments as a means of fixing winning gene combinations. In a compartmentalized system, the coevolution of hosts and parasites inevitably results in the onset of the arms race, one of the key factors in all biological evolution, which leads to a further increase in the complexity of both hosts and parasites, in particular through the evolution of defence and counter-defence systems (see below for more detailed discussion). Defence and counter-defence most probably are among the first (pre)biological functions.

With regard to the specific forms of compartmentalization, protective micro-environments, such as membrane vesicles resembling those emitted by some modern archaea and bacteria but initially with abiogenic membranes, also could have contributed to the compartmentalization, interaction and coevolution of genetic elements in the primordial pools, PMID: 18228159 (Gill, Forterre). Furthermore, although conventional thinking implies origin of virions (and hence bona fide viruses) after the advent of cells, the possibility exists that simple virions (capsids) antedate cells, having evolved as means of protection and dissemination of primordial genetic elements [63,64,70,71].

At present, the RNA world hypothesis appears to be the only plausible model for the transition from prebiotic to biological evolution [72,73]. In the RNA world, all reactions, including replication of RNA genomes, are postulated to have been catalysed by ribozymes. Given that parasites are inherent to replicator systems, they must have appeared already in the RNA world (incidentally, the Spiegelman experiments referred to above involve RNA genomes albeit protein-coding ones) leading to compartmentalization and complexification as outlined above. Mathematical modelling of replicator system evolution suggests that resilience to parasites increases when the information storage and catalytic functions are segregated, i.e. with the advent of dedicated operational devices such as proteins and dedicated information carriers such as DNA [68]. On the intuitive level, systems with separation of informational and operational functions and dedicated information careers would be more resistant to parasites than systems without such separation because parasites can only take over the operational but not the informational component, increasing the chance that the latter survives the invasion. Thus, the origin of translation (even though deciphering the concrete scenario in this case meets with great difficulty [74]) and the origin of DNA genomes, at least in part, were driven by the host–parasite arms race. Translation could emerge only in an ensemble of cooperators, given the requirement of multiple components [57]. The advent of translation thus gave an immediate advantage, in the form of highly efficient catalysts, to hosts in the race with parasites. However, the next step in the race would have been the adaptation of parasites to the new RNA-protein world, i.e. evolution of the ability to hijack host-produced proteins that could be employed for parasite replication, such as polymerases. The hosts responded by evolving enzyme specificity and compartmentalization (host genome replication catalysed *in cis*) to exclude parasites, and in response, some of the parasites captured host genes encoding proteins that could benefit the parasite replication, using the host translation machinery to make their own proteins.

The primordial genetic pool most probably evolved from the RNA-only stage (RNA world) towards a mixture of genetic elements with all replication–expression strategies that are employed by extant selfish elements [14,64]. For chemical reasons, the appearance of DNA is a pre-requisite of the evolution of larger genomes that eventually reached the chromosome size, further bootstrapping complexity and preparing the ground for the emergence of modern-type cells. The arms race between parasites and hosts in itself could have been a driving force behind the origin of DNA in parasites as a form of genome resistance to defence mechanisms evolved by hosts against RNA parasites [20,21,75,76].

DNA could not have emerged from the RNA world without reverse transcription. Retroelements, the most common selfish elements in modern eukaryotes that are widely represented also in bacteria and some archaea (even if less abundant than in eukaryotes), must have been among the first classes of elements that evolved in the primordial genetic pool after the advent of translation. Integration of such elements into host genomes must have coevolved with the increase in the size of DNA genomes, establishing one of the dominant trends in the host–parasite coevolution. Transposable elements with DNA genomes most probably evolved at the same stage. Genomic integration benefited the parasites providing them with a bet-hedging strategy, i.e. the ability to alternate passive propagation within the host genomes with autonomous reproduction [77,78]. However, the hosts also could extract benefits from this phenomenon through recruitment of selfish elements or their parts for host functions. This evolutionary strategy probably antedates modern-type cells and played important roles in evolutionary transitions (see below).

Evolutionary scenarios have been proposed in which DNA genomes originally evolved in viruses and subsequently took over the genetic system of RNA-based protocells [76,79]. As pointed out above, the origin of DNA in selfish elements is plausible in the context of the arms race in the primordial gene pool. However, the existence of RNA-only cells seems to meet substantial difficulties, which puts these hypotheses into question. A more parsimonious scenario includes DNA genomes already at an early stage of cell evolution.

The emergence of modern-type cells with membranes impermeable to polymers brought about a steep new barrier to the dissemination of parasites which thereafter needed to either spread via general routes of HGT or evolve special devices, such as virions (although see abovementioned one for the possibility of an even earlier evolution of virions), to survive outside host cells and infect new ones. Thus, the advent of modern-type cellular life forms caused differentiation of selfish elements into a continuum of forms that differ with respect to the cost to the host and transmissivity [40,41]. On one end of the spectrum are low cost–low transmissivity elements, such as low copy number plasmids, whereas on the other end are viruses that kill the host and transmit to a new one with high efficiency.

Thus, during the first two, arguably the most momentous evolutionary transitions in the evolution of life, coevolution of genetic parasites with their hosts caused dramatic complexification on both sides of the host–parasite divide.

## 3. The third major transition: origin of eukaryotic cells

After the origin of cells, the emergence of eukaryotic cells (eukaryogenesis) arguably involved the greatest jump in complexity that ever occurred in the course of the evolution of life [80–82]. Indeed, a typical eukaryotic cell is about 1000-fold larger than a typical archaeal or bacterial cell (by volume), contains an endomembrane system including membrane-bounded organelles such as mitochondria and the nucleus and is thoroughly compartmentalized such that free diffusion of polymers is strongly limited [83]. This dramatic rise in the organizational complexity of the cell is matched by increased genomic complexity [84,85]. Eukaryotes lack the tight coupling between the genome size and the number of protein-coding genes that is characteristic of prokaryotes, so eukaryotic genomes typically are (much) larger than prokaryotic genomes [84,86]. In some animals and plants, this mismatch reaches grotesque proportions such that protein-coding sequences account only for 1–2% of the genome [86,87]. Even more importantly, eukaryotes differ from prokaryotes in the major principle of gene architecture: eukaryotic genes are interrupted by multiple non-coding sequences, introns [87,88]. Introns are excised from the primary transcripts of eukaryotic genes and the flanking coding sequences (exons) are spliced by a highly complex molecular machine, the spliceosome. Although most unicellular eukaryote genomes are intron-poor, evolutionary reconstructions indicate that the last common ancestor of the extant eukaryotes was intron-rich, with 6–7 introns per gene, close to the intron density in modern vertebrates [89]. The exon–intron structure of the protein-coding genes makes a major contribution to the complexity of the eukaryotic proteomes through alternative splicing that yields multiple protein isoforms. On a different plane, the exon–intron architecture of eukaryotic genes appears to be linked to the organizational complexity of the eukaryotic cells. Splicing is tightly coupled with the nucleocytoplasmic export of mature mRNAs and the origin of the nucleus itself could have been driven by the necessity to prevent access to cytoplasm to immature, intron-containing transcripts that would be translated to yield aberrant, often toxic proteins, with catastrophic consequences to the cell [90–92].

All extant eukaryotes possess mitochondria or derivatives thereof, and under endosymbiotic scenarios of eukaryote origin [80], the prokaryote to eukaryote transition appears to have been driven by endosymbiosis between an α-proteobacterium, the future mitochondrion and a host cell that most probably was an archaeon of the Lokiarchaeota group [93–96]. The nature of the host of the proto-mitochondrion remains a subject of debate, with arguments presented in favour of the evolution of primitive eukaryotic cells prior to endosymbiosis [97,98]. The difficulties faced by these ‘protoeukaryotic’ evolutionary scenarios are twofold: first, primary amitochondrial eukaryotes have not been discovered despite considerable effort and so have to be postulated to be extinct; second, under these scenarios, there seems to be no plausible chain of causation for the origin of the complex features of the eukaryotic cellular organization. Although the debate on the origin of eukaryotes certainly is not over, the rest of the present discussion assumes the endosymbiotic model.

The domestication of the proto-mitochondrion apparently triggered an avalanche of events that in a rapid (on the evolutionary scale) succession led to the transition [91]. It appears likely that the primary trigger of the transformation of the cellular organization during eukaryogenesis was the dramatic boost to energy production provided by the (proto)mitochondria to the chimeric cell [85]. The increased energy production would result in the growth of the cell volume concomitant with a drop in the effective population size and resulting in reduced power of selection [99]. Under these conditions, slightly or even moderately deleterious mutations are fixed through genetic drift [100–102]. In the evolving chimeric cell, a dominant process was the integration of endosymbiont DNA into the host genome that was sustained by the constant lysis of the symbionts. Apparently, this unidirectional DNA flow involved extensive proliferation of Group II introns, bacterial retroelements that invaded the host genes in large numbers and gave rise both to the spliceosomal introns and the active RNA moieties of the spliceosome itself [91,103]. Strikingly, one of the central protein components of the spliceosome, Prp8, also is a Group II intron derivative, an inactivated reverse transcriptase (RT) [104]. Under this scenario, the origin of the spliceosome and the nucleus with its elaborate pore complexes that couple splicing with mRNA export effectively represents evolution of defence against deleterious effects of MGE. The evolution of certain other major functional systems of the eukaryotic cell, such as nonsense-mediated decay and the ubiquitin signalling network, also can be considered in the context of addition of extra layers of defence [91].

Importantly, in the case of eukaryogenesis, the invading MGE (Group II introns giving rise to the spliceosomal introns) apparently made a dual contribution to the complexity of the eukaryotic cell: first, by triggering the evolution of multiple, elaborate lines of defence, and second, by enabling alternative splicing. However, the contribution of bacterial retroelements to eukaryogenesis is not limited to these two major effects but additionally included linearization of the chromosomes [91]. The switch from circular to linear chromosomes appears to be a necessary condition to prevent the devastating genome instability that would have been caused by recombination between intron copies within a circular genome [105]. Replication of linear chromosomes requires copying of terminal repeats that is catalysed by the RT subunit of the telomerase, which was derived from the RT encoded by the same Group II introns, which were the ancestors of the spliceosomal introns and the key components of the spliceosome itself [106].

Remarkably, viral contribution was important also in the evolution of the mitochondria [107]. At an early stage of the eukaryotic evolution, although apparently after the divergence of the jacobids from the rest of eukaryotes, the typical bacterial multisubunit RNA polymerase was replaced by a single-subunit phage polymerase that is responsible for the transcription of the mitochondrial genomes in all eukaryotes apart from the excavates [108]. Apparently, at the same stage, the bacterial primase together with the replicative helicase were replaced by a single phage protein containing fused helicase and primase domains [109]. These replacements of key components of the mitochondrial replication and transcription machineries with the compact phage analogues seem to be an important aspect of the overall streamlining of information processing in the mitochondria.

The great majority of eukaryotes reproduce via regular sex, and the presence of dedicated meiotic genes in the genomes of even seemingly asexual forms implies that meiosis and sex are ancestral eukaryotic features. Hence, the origin of meiotic sex should be considered an intrinsic part of the major transition that led to the origin of eukaryotes. The origin and maintenance of meiosis and sex have been the subject of extensive theoretical analysis [110–112]. The principal cause of the near ubiquity of sex in eukaryotes seems to be the avoidance of Muller's ratchet (accumulation of deleterious mutations in a clonal population, leading to eventual mutational meltdown) through regular meiotic recombination [112–115]. This special mechanism of recombination could have become a necessity once HGT that performs the same function in prokaryotes [116] was curtailed by evolution of eukaryotic intracellular compartmentalization. Generation of diversity could be an extra benefit of sex [112]. However, these advantages readily explain the maintenance but not the origin of sex. Therefore, it has been proposed that sex originally evolved not because it is advantageous to the respective organisms, but under the pressure of MGE the spread of which is promoted by meiotic recombination [117,118]. Although this remains a hypothetical mechanism, the role of MGE in the evolution of meiosis is compatible with the observation that the archaeal homologues of the key enzymes of meiotic double-strand break repair, the nuclease Mre11 and the recombinogenic ATPase Rad50, form operons that are often located on plasmids or remnants of plasmids integrated in the chromosome [119]. Conceivably, at the early phase of the evolution of eukaryotes, when spread of plasmids via horizontal transfer was hampered, selection favoured highly recombinogenic variants of these repairs, which promoted the emergence of meiosis and with it their own spread. This scenario, although speculative, highlights a distinct and potentially important role of MGE in evolutionary transitions. In this case, in addition to the recruitment of MGE genes for a molecular function at the new level, a major component of the transition could have been driven by ‘selfish interests’ of MGEs.

Thus, selfish genetic elements were one of the key factors in the major evolutionary transition associated with the birth of eukaryotes to which they contributed both through the arms race that caused evolution of complex defence systems and by direct donation of essential parts of the eukaryotic cellular machinery.

## 4. The next major transition: origin of multicellularity

I skip the fourth major transition in Szathmáry's list [8], the acquisition of the cyanobacterial endosymbiont that became the chloroplast in the common ancestor of plants and algae. The next major transition is the evolution of multicellularity. Remarkably, unlike the preceding transitions, the origin of cells and eukaryogenesis, multicellularity is not a unique trait, but rather evolved independently on multiple occasions (hence, it is actually a series of similar transitions) [120–125]. Four of these involve the emergence of complex multicellular life forms, with marked cell and tissue differentiation, namely animals, plants, fungi and brown algae. Apart from these advanced forms, simpler versions of multicellularity, with some level of cell differentiation, evolved also in eukaryotes that are traditionally considered unicellular [126,127] and in certain groups of prokaryotes, such as cyanobacteria, actinomycetes and myxobacteria [128–130]. Moreover, many bacteria and archaea have the ability to self-organize into multicellular aggregates, in particular through quorum sensing or diffusion sensing [131,132]. The degree of cell specialization, if any, in such transient multicellular ensembles is not well understood.

One of the hallmarks of multicellular life forms is programmed cell death (PCD). PCD systems function in defence against pathogens and control of cell proliferation as well as at some stages of normal development [133–137]. In multicellular organisms, PCD appears ‘natural’, a mechanism that controls a lower organizational level, individual cells, for the benefit of the higher level, the organism, killing those cells that become harmful rather than useful to the organism. However, numerous, independent lines of evidence point to the existence of PCD also in many, probably most, unicellular life forms, which, at first glance, appears paradoxical [138–140]. Indeed, in multicellular organisms, PCD is ‘altruistic’ with respect to individual cells: some cells commit suicide so that other cells of the same organisms (and hence the organism as a whole) could live. This principle cannot work in unicellular life forms; altruistic traits can evolve only when there is selection at the level of cell ensembles, i.e. upon the transition to multicellularity. The driving forces behind such a transition are not obvious because generally the reproduction rate is highest in a free cell state (e.g. contact inhibition of cell culture growth). However, the situation changes when the cells are subject to pressure from pathogens. In this case, suicide of infected cells can protect neighbour cells in a multicellular aggregate and hence can be advantageous to the aggregate as a whole. Investigation of the phase space of an agent-based mathematical model of host–parasite coevolution has shown that increasing parasite pressure indeed leads to an increased advantage of cellular aggregates endowed with a PCD mechanism, and at some threshold pressure, this strategy becomes the only sustainable one [141]. The main conclusion of this modelling study is that multicellularity and PCD are inextricably coupled: one cannot evolve without the other. As with any altruistic behaviour, evolution of PCD is hampered by cheaters [142], in this case, cells that shed the suicide machinery but benefit from other cells committing suicide; proliferation of the cheaters can lead to the collapse of the entire aggregate. The threat posed by the cheaters can be overcome via kin selection (inclusive fitness) whereby microbial cells that form a multicellular aggregate are close relatives so that altruistic suicide effectively promotes the survival of the genotype of the dying cells [143–145]. Kin selection is likely to have been essential in the pathogen-driven evolution of multicellularity.

Given the ubiquity of PCD, in particular in the form of prokaryotic toxin–antitoxin (TA) systems [146–149], it appears most likely that this phenomenon evolved concomitantly with or at least shortly after the very first cells. Accordingly, simple forms of multicellularity, aggregation of cells with little or no differentiation, are most probably (nearly) as old as cellular life itself. The evolutionary coupling of PCD with multicellularity presents an appearance of a chicken-or-egg paradox: PCD cannot evolve without multicellularity, but multicellularity does not appear to be evolvable in the absence of the fitness gain provided by PCD under the pressure of parasites. The solution is suggested by the properties of the TA systems, which are partly selfish genetic elements that are addictive to cells that harbour them ([150–152]; see discussion below). The TA systems could have evolved as plasmid addiction modules in unicellular organisms and then recruited by aggregating microbes as ready-made PCD devices [141].

Thus, the host–pathogen arms race probably was one of the key factors behind the evolution of simple multicellularity, which prepared the ground for the emergence of advanced multicellular organisms, on several independent occasions. It appears likely that the origin of animals and plants involved bursts of MGE transposition that accelerated evolution but, unfortunately, the details of these key stages of eukaryotic evolution are difficult to reconstruct. Nevertheless, at least one case in point is striking: the origin of the Hedgehog proteins, key regulators of animal development, from inteins, a peculiar class of parasites that combine transposition at the DNA level with splicing at the protein level.

## 5. Evolution of defence systems: a hallmark of evolutionary transitions

The ubiquity and abundance of genetic parasites along with the presence of defence systems in (nearly) all cellular life forms strongly suggest that their coevolution had been one of the key aspects of the evolution of life at least since the emergence of the protocells but possibly, even since the pre-cellular stage, i.e. through all the major transitions. The startling and essential feature of defence systems, especially in prokaryotes, is that they themselves possess properties of selfish elements and/or are derived from such elements [152–156]. The selfish character of defence systems is manifested in the addictiveness of the simplest of them, namely prokaryotic TA, abortive infection (AI) and restriction-modification (R-M) modules, to the cells in which they reside [152]. The molecular mechanisms of TA (type II), AI and R-M differ in details but the central principle is one and the same. Each of these systems consists of two (or less often, three) proteins encoded by closely spaced, co-regulated genes. The mechanism of the TA and related AI systems is simple and elegant [146,148,155]. One of the two proteins is a toxin that kills the cell unless complexed with the second protein, the antitoxin. The antitoxin is much less stable than the toxin and therefore, when the TA/AI genes are lost or inactivated, the toxin is released and the cell dies, hence the addictiveness of the TA/AI systems that often reside on plasmids, which the TA endow with addictiveness and exploit as vehicles for horizontal spread. Thus, the TA/AI systems actually represent a special class of MGE (or perhaps more precisely, ‘quasi-MGE’). Although they do not encode any information to direct their own replication, they do promote their own mobility by hitchhiking on plasmids and making host cells maintain those plasmids.

The defence function of the TA and AI is realized via altruistic PCD, and the simplicity of these systems is compatible with their emergence being concomitant with the origin of protocells that, under the concept developed here, were already prone to aggregation (primitive multicellularity). Toxins possess several different activities but the most common one is that of interferase, an RNAse that cleaves mRNAs within the ribosome [157,158]. The interferases are small proteins with a simple fold that are likely to have been among the earliest enzymes to evolve. Another common, still insufficiently characterized toxin component of TA and AI systems is a nucleotidyltransferase, another mimimal-sized, possibly primitive enzyme [147,159].

The R-M systems differ from the TA with regard to the principle of their protective action: they directly attack invading viruses and other foreign DNA rather than killing the infected cell [160,161]. Traditionally, R-M systems are not considered as toxins–antitoxins but in effect they function on a similar principle. In this case, the toxin is a DNA endonuclease and the antitoxin is a methylase that renders the host DNA resistant to the endonuclease. The methylase and the endonuclease share strict sequence specificity, so when the methylase is inactivated, the endonuclease becomes toxic and kills the cell. Moreover, at least some R-M modules are addictive. Although less thoroughly understood than the TA case, it has been shown that when an R-M module is lost from a cell, the methylase activity drops faster than the endonuclease activity resulting in the so-called post-segregational cell killing [154,162,163]. The R-M modules are often transferred on plasmids, transposons and viruses, and effectively belong to the same class of ‘quasi-MGE’ as the TA and AI systems.

Although the simplest defence systems, TA, AI and R-M, that collectively represent innate immunity in prokaryotes, possess properties of MGE, the more complex systems of adaptive immunity, in particular CRISPR-Cas, do not seem to show such features directly [164,165]. Although horizontal transfer of CRISPR-Cas systems is common, they do not appear to be addictive. However, the origin of adaptive immunity, which itself can be considered a minor evolutionary transition, involved substantial contributions from MGE. Specifically, the CRISPR-Cas system is thought to have evolved through recombination of a self-synthesizing transposon of the casposon family (so named because the integrase/transposase of these elements is a homologue of the Cas1 protein, the key enzyme at the adaptation stage of the CRISPR-Cas response [166]) with a hypothetical ancestral innate immunity locus [167]. Indeed, the reactions catalysed by an integrase during transposon integration and by Cas1 during CRISPR-Cas adaptation are mechanistically nearly identical [168]. Transposons appear to be perfect, ‘pre-manufactured’ tools for recruitment by evolving adaptive immunity systems and potentially other mechanisms of genome manipulation. This feature of transposons is strikingly demonstrated by the parallel evolution of CRISPR-Cas, vertebrate adaptive immunity and the system of DNA elimination in ciliates [167,169]. In all three cases, unrelated transposons appear to have donated not only the enzyme catalysing genome rearrangement, but also the repeats that serve as the recognition sites for this enzyme. Remarkably, a different group of transposons made further contributions to the evolution of prokaryotic adaptive immunity, replacing the ancestral effector modules of Class 1 CRISPR-Cas systems with transposon-derived nucleases [170]. As a result, Class 2 CRISPR-Cas systems are fully derived from MGE.

Most if not all of the defence systems in prokaryotes are ‘guns for hire’ that can function either as defence weapons for cells against parasites or counter-defence (that is, actually, offence) weapons employed by parasites against the hosts [23]. This duality extends from the simplest defence modules such as TA and R-M to the complex CRISPR-Cas immune systems. The defence and counter-defence weapons, at least in some case, clash directly. As a case in point, it has been shown that a phage-encoded CRISPR-Cas system abrogates the activity of host R-M systems, thus enabling phage reproduction [171].

The semi-selfish defence systems based on the addiction principle have not survived the prokaryote to eukaryote transition, presumably because of the irreversible decay of operons in eukaryotes, which made the toxin components of such systems highly deleterious [172]. Remarkably, however, components of these systems have been extensively recruited as eukaryotic signalling proteins and chromatin remodelling-modification enzymes [173].

The key aspect of anti-parasite defence evolution is quite simple: the active moieties of defence systems are weapons capable (if unchecked) of destroying genomes. These weapons can be turned against a parasite or against its host and also have the potential to evolve into selfish elements in their own right. In their simplest form, such weapons, simply put, nucleases, most probably, evolved already in the primordial, pre-cellular genetic pool. Their integration into more complex (counter-)defence systems apparently tracked evolutionary transitions and itself represented a series of minor transitions. Taken together, all these observations show that evolution of defence systems is intrinsically intertwined with the evolution of the selfish genetic elements. The link between the two is not limited to the obvious effects of the arms race but also involves perpetual exchange of components. As discussed above, MGE appear to have played key roles in several major evolutionary transitions and this involvement is in large part realized through the evolution of defence systems.

## 6. Concluding remarks

The biosphere is literally dominated by viruses and other MGE. Emergence of parasites is an inherent property of replicator systems. These two facts predicate the multiple and essential roles of MGE in the evolution of life. Here, I identify specific contributions of selfish elements to the major evolutionary transitions. Such contributions come in two forms: the arms race that triggers major innovations in cellular life forms (hosts of the selfish elements) and direct recruitment of parts of selfish elements for key cellular functions. At the core of each evolutionary transition is the emergence of a new level of cooperation between biological entities and hence of a new level of selection (figure 1). One of the key driving forces in the evolution of cooperation—and of compartmentalization that precedes and facilitates the emergence of collectives—is defence against parasites. An ensemble can make use of mechanisms that are inaccessible to individual entities. These are the mechanisms that involve an altruistic component and allow the collective to avoid collapse under parasite pressure, stabilizing the host–parasite system as a whole. A complementary aspect of host–parasite coevolution is the ‘guns for hire’ gamut whereby the same molecular systems are employed by hosts and parasites for defence and counter-defence, respectively. These molecules are tools of genome manipulation and in that capacity reach beyond straightforward defence as demonstrated by many cases of exaptation such as eukaryotic telomerase, PRP8 and various TA-derived proteins involved in signalling and chromatin remodelling. Conversely, defence is not limited to the recruitment of ‘genomic weapons’ and can be asymmetrical as implied by the models of the nucleus origin.

I left three of the seven transitions in Szathmáry's list out of this discussion [8] (table 1), namely the origin of photosynthetic eukaryotes that was precipitated by the symbiosis between the ancestor of Archaeplastida, the origin of eusociality that occurred independently in two groups of animals, insects and mammals, and the origin of language-endowed (human) society. Before closing, a few words about these transitions are due. The genomes of many cyanobacteria contain Group II introns, which have proliferated in chloroplast genomes [174,175]. Thus, after the cyanobacterial endosymbiosis, the host genome was subject to an attack by these elements, analogous to the case of the mitochondria. Notably, the chloroplast-derived genes have only very slightly lower intron density than ancestral plant genes, i.e. are almost saturated with introns [176]. This accumulation of introns certainly played an important role in the assimilation of the chloroplast-derived genes by plant cells. However, it is unclear whether cyanobacterial Group II introns gave rise to these introns, and, more generally, so far imprints of cyanobacterial intron attack in plant or algal genomes have not been detected. Thus, the role of MGE in the photosynthetic transition, if any, remains uncertain.

The origin of eusociality and especially the origin of societies endowed with language, while formally fitting the definition of a transition, appear fundamentally different from other transitions in that they involve mainly changes in the behaviour of macro-organisms as opposed to molecular innovations implicated in the preceding transitions (even though limited, highly specific molecular changes certainly were involved in the last two transitions as well). Nevertheless, a role of virus infections or transposon mobilization in the evolution of social animals is imaginable and could be a subject of interesting research. For instance, it is imaginable that infections could be a driving force in the evolution of insect-type eusociality, where reproduction is limited to specialized individuals such as queens that could be specifically protected. Apart from such putative involvement of genetic parasites, susceptibility to other varieties of parasites, e.g. animal ectoparasites, is likely to be an important factor in the evolution of eusociality [177,178].

The contribution of MGE to the evolution of cellular organisms is by no account limited to the major transitions. Both diversification of defence and counter-defence mechanisms and exaptation of MGE for cellular functions occur continuously throughout the course of evolution [179]. The origin of the vertebrate adaptive immunity in which a Transib family transposon played a central role [180,181] is mentioned above. Another striking example is the repeated exaptation of envelope protein genes of retroviruses that gave rise to syncytins, the mammalian placental receptors [182–184]. This case parallels the ‘guns for hire’ theme because the hosts exploit the fusogenic properties of the Env proteins used by the viruses to infect the host cells.

Clearly, defence against parasites is not the only factor behind the evolutionary transitions. There is at least one other powerful force driving the evolution of cooperation, namely differential production of ‘public goods’ by individuals that benefits an ensemble [185,186]. The public goods paradigm applies to all stages in the evolution of life, including each of the major transitions. The production of such goods could have driven the origin of the first cooperatives of primordial genetic elements [57] and is also relevant for the endosymbiotic transitions in which endosymbionts and hosts supply complementary sets of metabolites and proteins [187,188]. Importantly, anti-parasite defence and ‘public goods’ sharing are often directly linked. Destruction of parasites as well as altruistic suicide, on the one hand, protect the host population; and on the other hand, result in production of consumables such as nucleotides and amino acids. Therefore, differentiation between the factors that drive cooperation between individuals and, hence, an evolutionary transition is not always meaningful: perhaps, more often than not, both drivers are in action. Together, these two complementary and interacting factors, host–parasite coevolution and production and sharing of resources (public goods), could account for many if not most aspects of evolutionary transitions (figure 1).
